# A machine learning model for distinguishing Kawasaki disease from sepsis

**DOI:** 10.1038/s41598-023-39745-8

**Published:** 2023-08-02

**Authors:** Chi Li, Yu-chen Liu, De-ran Zhang, Yan-xun Han, Bang-jie Chen, Yun Long, Cheng Wu

**Affiliations:** 1 Department of Gastroenterology, Children’s Hospital of Anhui Medical University, The Fifth Clinical Medical College of Anhui Medical University, Hefei, 230000 Anhui China; 2 Department of Otolaryngology, Head and Neck Surgery, The First Affiliated Hospital of Anhui Medical University, Anhui Medical University, Hefei, 230000 Anhui China; 3 Department of Neurosurgery, The Second Affiliated Hospital of Anhui Medical University, Anhui Medical University, Hefei, 230000 Anhui China; 4 Department of Oncology, The First Affiliated Hospital of Anhui Medical University, Anhui Medical University, Hefei, 230000 Anhui China

**Keywords:** Paediatric research, Rheumatic diseases, Risk factors, Diagnostic markers

## Abstract

KD is an acute systemic vasculitis that most commonly affects children under 5 years old. Sepsis is a systemic inflammatory response syndrome caused by infection. The main clinical manifestations of both are fever, and laboratory tests include elevated WBC count, C-reactive protein, and procalcitonin. However, the two treatments are very different. Therefore, it is necessary to establish a dynamic nomogram based on clinical data to help clinicians make timely diagnoses and decision-making. In this study, we analyzed 299 KD patients and 309 sepsis patients. We collected patients' age, sex, height, weight, BMI, and 33 biological parameters of a routine blood test. After dividing the patients into a training set and validation set, the least absolute shrinkage and selection operator method, support vector machine and receiver operating characteristic curve were used to select significant factors and construct the nomogram. The performance of the nomogram was evaluated by discrimination and calibration. The decision curve analysis was used to assess the clinical usefulness of the nomogram. This nomogram shows that height, WBC, monocyte, eosinophil, lymphocyte to monocyte count ratio (LMR), PA, GGT and platelet are independent predictors of the KD diagnostic model. The c-index of the nomogram in the training set and validation is 0.926 and 0.878, which describes good discrimination. The nomogram is well calibrated. The decision curve analysis showed that the nomogram has better clinical application value and decision-making assistance ability. The nomogram has good performance of distinguishing KD from sepsis and is helpful for clinical pediatricians to make early clinical decisions.

## Introduction

Kawasaki disease (KD), also known as mucocutaneous lymph node syndrome, is most common in children under 5 years old. It is an acute systemic vasculitis that mainly damages small and medium-sized blood vessels. Tomisaku Kawasaki first reported this in 1967^1,2^. The prevalence of KD varies widely across ethnic groups and currently ranges from 4–25/100,000 in children under 5 years old in North America, Australia, and Europe^3–5^. Compared to the United States and Europe, the incidence is 10–20 times higher in Japan, Korea, and Taiwan^6–8^. Additional studies have shown that the incidence of KD continues to rise in Asia^9–11^. The main clinical features of KD are fever, bilateral nonexudative conjunctivitis, oral changes, erythematous rashes (appear in the acute phase of the disease and affects 80–90% of patients with KD), extremity changes (appear in the acute and subacute phases of the disease and affect 80–90% of patients with KD), and cervical lymphadenopathy (appear in the acute phase of the disease and affects 50–60% of patients with KD)^12,13^. The studies showed that, except for the fever, conjunctivitis was the most frequent in children with KD, while cervical lymphadenopathy was the least frequent in children with KD^14,15^. Children who do not meet sufficient primary clinical presentations are diagnosed with incomplete KD^12^. The incidence of major clinical manifestations of incomplete KD, except for the fever, was less than that of complete KD, while cervical lymphadenopathy was the most pronounced^14,15^. The prevalence of incomplete KD varies from region to region, ranging from 16.1 to 48.4%^16–19^. Yahui et al. found that the incidence of both complete and incomplete KD increased over time, but that the incidence of incomplete KD increased more rapidly than that of complete KD (0.35 cases per 100,000 per year for incomplete KD compared to complete KD)^20^. The main complication of KD is coronary artery abnormalities (CAL), but additional coronary complications may also occur. The incidence of coronary artery aneurysms is about 20–30% in untreated cases^21,22^. Children younger than 6 months tend to have incomplete KD, which often delays their diagnosis and treatment^23^. These patients tend to be at high risk for CAL and intravenous immunoglobulin (IVIG) resistance^12,23–25^. Adults with coronary artery diseases are usually diagnosed retrospectively with incomplete KD^26,27^. However, the etiology of KD is still unknown. Based on multiple studies, the consensus among clinical researchers is that KD is an immune-mediated disease caused by an infection in patients with genetic predisposition^28–31^. Currently, in the absence of etiological detection, we diagnose KD mainly on the basis of clinical manifestations and the exclusion of other known pathogenic diseases with similar clinical manifestations to KD. A systematic review by the Cochrane Collaboration showed that a single dose of 2 g/kg IVIG administered before day 10 after onset reduces the development of CAL^32^. Therefore, according to current guidelines, high-dose IVIG remains the first-line treatment for KD^12^. However, delayed or missed diagnosis of KD in clinical practice may also lead to over-medication and even invasive treatment of children with KD^33^. Therefore, early and correct diagnosis and treatment of KD are crucial for prognosis.

Sepsis is a systemic inflammatory response syndrome caused by infection and a significant cause of septic shock and multiple organ dysfunction syndromes. In high-income countries, more than 4% of hospitalized children and 8% of PICU children suffer from sepsis^34–38^. Mortality in children with sepsis ranges from 4 to 50%, depending on disease severity, risk factors, and geographic location^34,35,39–41^. Therefore, early diagnosis and appropriate treatment are essential to optimize outcomes in children with sepsis.

Common symptoms of sepsis in children include fever, tachycardia, changed mental status and disturbed inflammatory factors^42^. Reyes et al. reported that 20 patients with sepsis had an average WBC of 11.02 × 10^9^/L, an average absolute neutrophil count of 6.84 × 10^9^/L and a mean CRP of 66.80 mg/L^43^. Man Man Niu et al. showed that PCT was significantly elevated in patients with sepsis^44^. However, sepsis has a non-specific and diverse clinical presentation, and a simplistic and objective definition to contain the entire spectrum of sepsis has become an excellent task^45^. The clinical manifestations of sepsis in neonates and children can often be so vague that they are challenging to identify^42^. These exacerbate the difficulty of sepsis diagnosis. In the early stages of the disease, KD patients have an acute onset of hyperthermia, decreased general condition and cooperativeness, increased heart rate, and some or all of the clinical manifestations of the primary diagnosis as the disease progresses^46,47^. Moreover, inflammatory markers such as WBC, absolute neutrophil, CRP, and PCT were also remarkably elevated in KD^44^. KD and sepsis share similarities in both clinical presentation and tests for inflammatory markers. However, the two treatment modalities are different, making the differential diagnosis of the two particularly important.

Harada's score was initially used to determine whether IVIG should be administered to patients with KD^48^. It is now also used in some medical centers to predict the risk of the coronary aneurysm in KD patients. As the understanding of the disease increased, in 2006, Kobayashi et al. proposed a new risk score to predict the risk of CALs in patients with KD^49^. However, few studies have been done to discriminate KD from sepsis in children^50^. Therefore, there is a need to screen for independent predictors that distinguish KD from sepsis to help us in early therapeutic interventions and improve prognosis in the clinic. Advances in machine learning and statistical methods have helped us develop clinical predictive models and assist physicians in making rapid diagnoses. Hence, the main objective of this study was to use the screened risk factors to develop and validate a machine learning model to differentiate KD and sepsis to assist pediatricians in making a rapid and accurate judgment.

## Results

### Characteristics of the overall patients

Our study recruited 309 sepsis patients and 299 KD patients, including 22 patients with incomplete KD and 2 with Kawasaki disease shock syndrome (KDSS). 221 sepsis and 205 KD formed our training set (N = 426), and 88 sepsis and 94 KD formed our validation set (N = 182). The baseline characteristics between the KD and sepsis groups are shown in Table S1 of Supplemental Material. The results of comparing KD patients and sepsis patients in the training and test sets are shown in Table 1. There was a significant difference in weight, WBC, neutrophil, lymphocyte count, monocyte, eosinophil, LMR, platelet to lymphocyte count ratio (PLR), albumin (ALB), albumin/globulin (AGR), prealbumin (PA), hematocrit (HCT), platelet (PLT), red blood cell count (RBC), alanine aminotransferase (ALT), γ-glutamyltransferase (GGT), sodium (Na), blood urea Nitrogen (BUN), calcium (Ca) (p < 0.05) between the KD patients and the septic patients in both the training and test groups. In addition, the differences in age, height, red blood cell distribution width (RDW), and total protein (TP) in the training set were statistically significant, so they were included in the variable selection.

**Table 1:** The results of comparing KD patients and sepsis patients in the training set and validation set.

Variables	Training set			Validation set		
	Sepsis	KD	p	Sepsis	KD	p
Age (months)	26.5 ± 25.7	29.18 ± 22.51	0.004	24.55 ± 23.26	26.59 ± 19.77	0.096
Height (cm)	84.83 ± 21.27	88.89 ± 16.95	0.002	84.99 ± 20.76	87.66 ± 15.72	0.133
Weight (kg)	13.03 ± 6.97	13.55 ± 4.91	0.004	12.77 ± 7.58	13.19 ± 4.51	0.036
BMI (kg/m^2^)	17.5 ± 3.44	16.93 ± 2.5	0.167	16.76 ± 2.40	17.04 ± 2.4	0.391
WBC (× 10^9^/L)	21.52 ± 9.36	15.13 ± 5.79	< 0.001	21.46 ± 7.38	15.53 ± 4.69	< 0.001
Neutrophil (× 10^9^/L)	14.44 ± 7.64	10.4 ± 5.16	< 0.001	14.34 ± 6.86	10.59 ± 4.27	< 0.001
Lymphocyte (× 10^9^/L)	5.15 ± 5.39	3.5 ± 2.04	< 0.001	4.95 ± 2.64	3.73 ± 2.27	< 0.001
Monocyte (× 10^9^/L)	2.44 ± 9.16	0.92 ± 0.51	< 0.001	1.95 ± 1.09	0.9 ± 0.46	< 0.001
Eosinophil (× 10^9^/L)	0.11 ± 0.2	0.31 ± 0.44	< 0.001	0.11 ± 0.20	0.32 ± 0.39	< 0.001
NLR	4.57 ± 4.95	4.13 ± 3.24	0.536	4.3 ± 4.19	4.41 ± 4.16	0.651
PLR	94.39 ± 74.49	135.73 ± 85.22	< 0.001	81.43 ± 59.36	129.14 ± 83.96	< 0.001
LMR	3.28 ± 3.27	4.47 ± 2.86	< 0.001	3.33 ± 2.87	4.84 ± 3.4	< 0.001
RBC (× 10^12^/L)	4.23 ± 0.51	4.09 ± 0.41	0.001	4.24 ± 0.5	4.1 ± 0.41	0.008
HB (g/L)	110.04 ± 15.51	108.38 ± 10.84	0.107	112.33 ± 12.92	108 ± 10.86	0.016
HCT (%)	34.24 ± 3.99	33.44 ± 3.3	0.043	35.2 ± 3.82	33.13 ± 3.38	< 0.001
MCV (fL)	80.21 ± 6.87	81.36 ± 5.45	0.063	82.25 ± 5.61	80.23 ± 4.57	0.009
MCHC (g/L)	322.67 ± 15.59	324.47 ± 15.77	0.658	319.15 ± 13.31	326.38 ± 13.42	< 0.001
RDW (%)	11.17 ± 2.68	11.87 ± 2.78	0.007	11.1 ± 2.68	11.97 ± 2.79	0.103
PLT (× 10^9^/L)	326.48 ± 122.61	367.06 ± 134.58	0.001	308.19 ± 118.24	366.88 ± 149.23	0.012
TBIL (μmol/L)	8 ± 4.74	10.28 ± 10.73	0.310	8.82 ± 6.84	10.94 ± 14.12	0.658
DBIL (μmol/L)	3.32 ± 2.15	5.64 ± 8.77	0.104	3.72 ± 3	6.22 ± 11.52	0.532
IBIL (μmol/L)	4.69 ± 2.84	4.65 ± 2.88	0.854	5.11 ± 4.39	4.71 ± 3.02	0.900
TP (g/L)	63.8 ± 7.71	61.89 ± 6.49	< 0.001	63.65 ± 5.96	62.73 ± 6.9	0.185
ALB (g/L)	40.79 ± 5.04	36.75 ± 4.38	< 0.001	40.18 ± 4.49	37.04 ± 4.02	< 0.001
GLB (g/L)	23.44 ± 4.74	25.15 ± 4.86	< 0.001	23.47 ± 4.54	25.7 ± 6.31	0.003
AGR	1.91 ± 1.57	1.51 ± 0.32	< 0.001	1.76 ± 0.37	1.5 ± 0.31	< 0.001
PA (g/L)	123.28 ± 46.67	89.19 ± 31.02	< 0.001	119.99 ± 43.74	89.84 ± 29	< 0.001
ALT (U/L)	27.71 ± 85.34	75.08 ± 125.51	< 0.001	31.75 ± 71.58	65.97 ± 108.88	< 0.001
AST (U/L)	41.17 ± 130.22	60.34 ± 111.98	0.721	45.04 ± 105.58	67.78 ± 182.11	0.827
ALP (U/L)	145.39 ± 45.62	143.87 ± 59.3	0.077	150.78 ± 49.11	157.61 ± 95.31	0.715
GGT (U/L)	18.57 ± 20.7	69.5 ± 90.55	< 0.001	27.12 ± 59.6	65.46 ± 87.69	< 0.001
LDH (U/L)	308.54 ± 266.75	278.69 ± 81.82	0.091	334.89 ± 273.21	295.9 ± 127.81	0.192
BUN (mmol/L)	4 ± 3.27	4.92 ± 19.39	0.018	4.25 ± 4.42	3.12 ± 1.35	< 0.001
Na (mmol/L)	136.58 ± 3.31	135.73 ± 2.79	0.001	136.6 ± 3.13	135.81 ± 2.75	0.071
Ca (mmol/L)	2.9 ± 6.83	2.33 ± 0.16	< 0.001	2.43 ± 0.17	2.35 ± 0.13	< 0.001
Fe (mmol/L)	3.2 ± 2.4	3.15 ± 2.43	0.287	2.96 ± 2.00	3.2 ± 1.98	0.041
CRP (mg/L)	84.41 ± 60.01	83.74 ± 8.59	0.883	88.01 ± 51.26	73.63 ± 51.14	0.012
Gender						
Female	89 (40.3%)	70 (34.1%)	0.228	34 (38.6%)	30(31.9%)	0.427
Male	132 (59.7%)	135 (65.9%)		54 (61.4%)	64 (68.1%)	

### Characteristics of training and validation sets

70% of patients were randomly assigned to the training set and the remaining 30% to the validation set. There is no significant difference between the training set and the validation set of variables (p > 0.05), which means that the difference between the training set and the testing set is slight, and the model is stable (Table 2).

**Table 2:** Comparisons of the KD and sepsis group between the training set and validation set.

Variables	Training data set	Validation data set	*P-*value
Outcome			
Sepsis	221 (51.9%)	88 (48.4%)	0.479
KD	205 (48.1%)	94 (51.6%)	
Height (cm)	86.78 ± 19.4	86.37 ± 18.33	0.892
Age (months)	27.56 ± 22.17	28.45 ± 21.47	0.564
Weight (kg)	13.28 ± 6.07	12.99 ± 6.17	0.604
BMI (kg/m^2^ )	17.23 ± 3.03	16.91 ± 2.4	0.393
WBC (× 10^9^/L)	18.44 ± 8.46	18.4 ± 6.81	0.752
Neutrophil (× 10^9^/L)	12.49 ± 6.86	12.4 ± 5.96	0.643
Lymphocyte (× 10^9^/L)	4.36 ± 4.21	4.32 ± 2.52	0.355
Monocyte (× 10^9^/L)	1.71 ± 6.64	1.41 ± 0.98	0.809
Eosinophil (× 10^9^/L)	0.21 ± 0.35	0.22 ± 0.32	0.273
NLR (× 10^9^/L)	4.36 ± 4.21	4.35 ± 4.17	0.929
PLR	114.28 ± 82.38	106.08 ± 76.73	0.230
LMR	3.85 ± 3.13	4.11 ± 3.24	0.617
RBC (× 10^12^/L)	4.17 ± 0.47	4.17 ± 0.46	0.703
HB (g/L)	109.24 ± 13.48	110.09 ± 12.06	0.398
HCT (%)	33.85 ± 3.7	34.13 ± 3.74	0.323
MCV (fL)	80.76 ± 6.25	81.21 ± 5.18	0.706
MCHC (g/L)	323.54 ± 15.69	322.88 ± 13.81	0.511
RDW (%)	11.51 ± 2.75	11.55 ± 2.76	0.632
PLT (× 10^9^/L)	346.01 ± 129.95	338.51 ± 137.94	0.510
TBIL (umol/L)	9.1 ± 8.26	9.92 ± 11.23	0.756
DBIL (umol/L)	4.43 ± 6.38	5.01 ± 8.61	0.720
IBIL (umol/L)	4.67 ± 2.86	4.9 ± 3.74	0.852
TP (g/L)	62.88 ± 7.2	63.18 ± 6.46	0.813
ALB (g/L)	38.85 ± 5.14	38.56 ± 4.53	0.458
GLB (g/L)	24.26 ± 4.87	24.62 ± 5.62	0.607
AGR	1.72 ± 1.17	1.63 ± 0.36	0.413
PA (g/L)	106.87 ± 43.36	104.41 ± 39.75	0.558
ALT (U/L)	50.5 ± 109.05	49.43 ± 94.06	0.884
AST (U/L)	50.39 ± 122.02	56.79 ± 150.09	0.627
ALP (U/L)	144.66 ± 52.59	154.31 ± 76.41	0.069
GGT (U/L)	43.08 ± 69.33	46.92 ± 77.63	0.646
LDH (U/L)	294.18 ± 200.68	314.79 ± 211.31	0.069
BUN (mmol/L)	4.45 ± 13.64	3.67 ± 3.26	0.822
Na (mmol/L)	136.17 ± 3.1	136.19 ± 2.96	0.921
Ca (mmol/L)	2.63 ± 4.93	2.39 ± 0.16	0.924
Fe (mmol/L)	3.18 ± 2.42	3.08 ± 1.99	0.530
CRP (mg/L)	84.09 ± 59.26	80.58 ± 51.56	0.633
Gender			
Female	159 (37.3%)	64 (35.2%)	0.679
Male	267 (62.7%)	118 (64.8%)	

### Risk factors selection for construction of the nomogram

**Figure 1:**
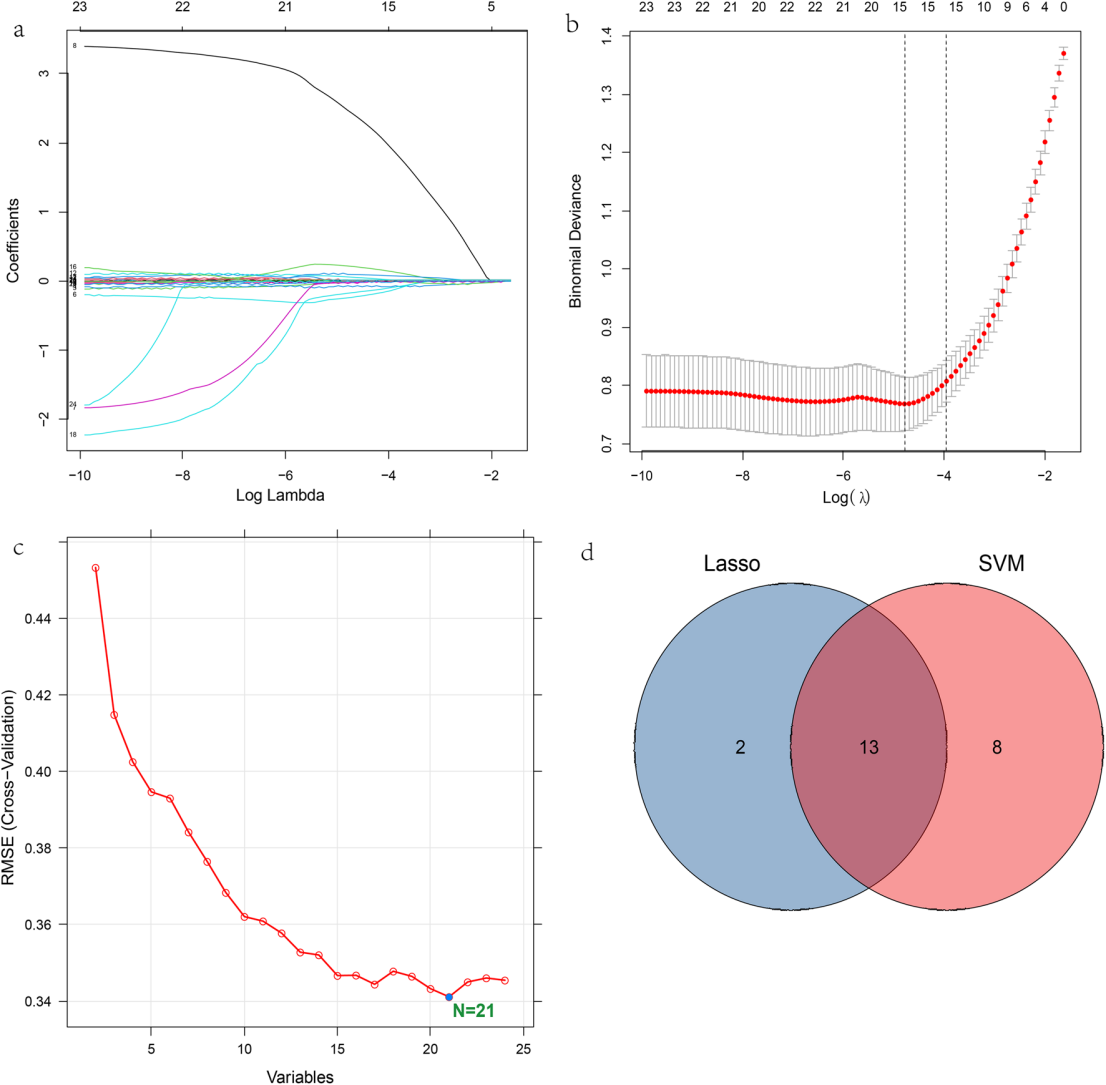
(**a**) LASSO coefficient profiles of the candidate predictors. (**b**) Selection of the optimal penalization coefficient in the LASSO regression. (**c**) The results of support vector machine. (**d**) To show the results of the intersection of SVM and LASSO using Venn diagram.

**Figure 2:**
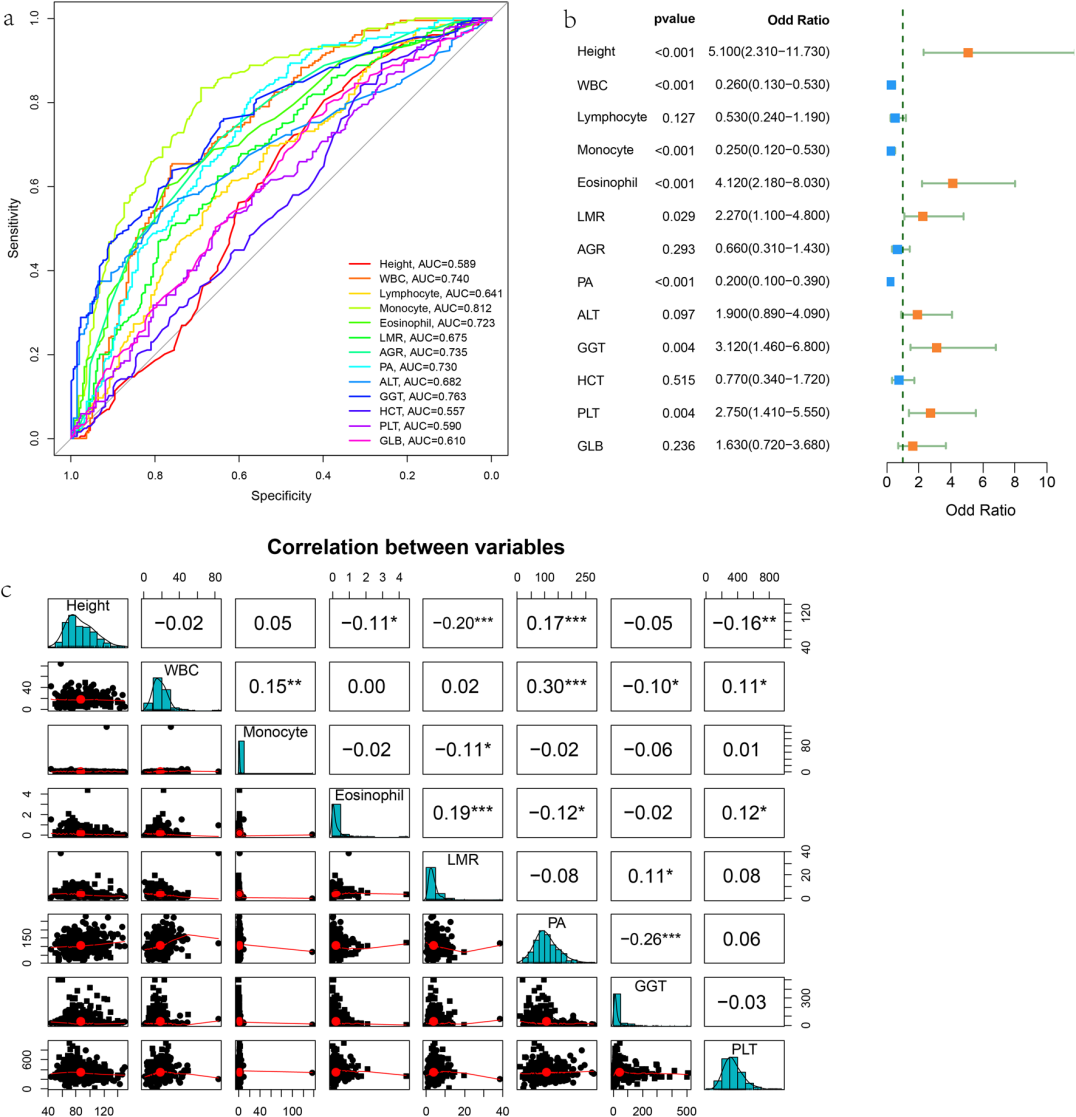
(**a**) Receiver operating characteristic (ROC) curves of 13 continuous variables. (**b**) The results of forest plot. (**c**) The diagonal line shows the distribution of the variables itself. The lower triangle shows scatter plot of matrices with bivariate scatter plots. The numbers on the upper triangle indicate the Pearson correlation coefficient and the stars indicate the degree of significance.

**Figure 3:**
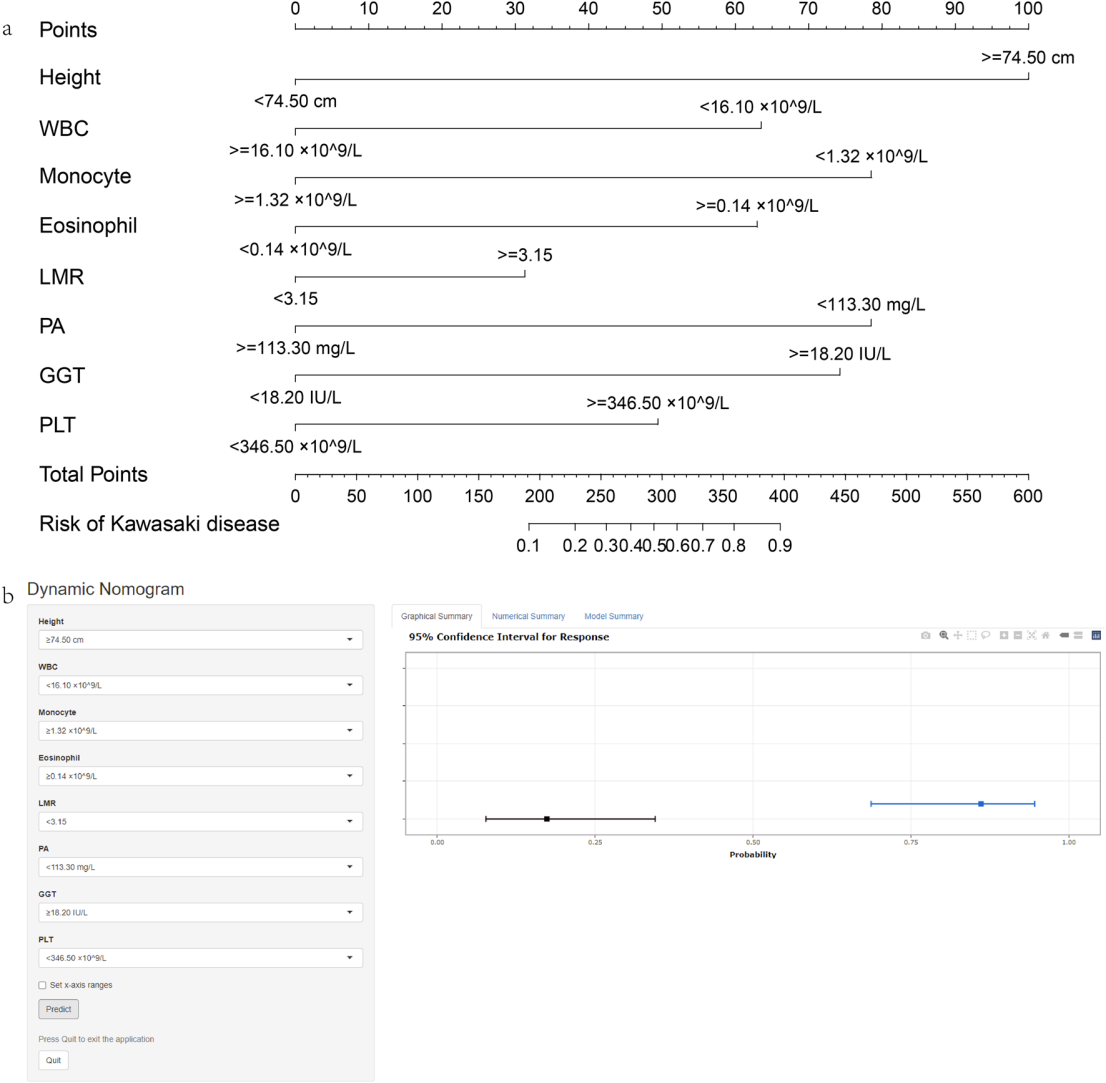
(**a**) The nomogram prediction score of Kawasaki disease in the differentiation of sepsis. (**b**) Online dynamic nomogram accessible at https://hanchenchen.shinyapps.io/KD-nom/.

Initially, there were 24 variables with statistically significant differences, and then the data from the training set was screened for predictors. Twenty-four variables were reduced to 15 potential predictors using the least absolute shrinkage and selection operator (LASSO) (Fig. 1a,b) and 21 using support vector machine (SVM) (Fig. 1c). Using SVM and LASSO to take the intersection (Venn diagram) (Fig. 1d), 13 variables are used to build the model. We converted these 13 continuous variables into categorical variables based on the cut-off value at the maximum area under the curve (AUC) (Fig. 2a and Table 3) and built a multiple logistic regression model. The forest plot (Fig. 2b) and Table 4 show that the final logistic regression model contains 8 independent predictors. Collinearity between different variables is represented in Fig. 2c. According to logistic regression results, a nomogram (Fig. 3a) was created online at https://hanchenchen.shinyapps.io/KD-nom/ (Fig. 3b).

**Table 3:** The results of receiver operating characteristic (ROC) curves of 13 continuous variables.

Variables	AUC	Cut-off value	Youden index	Sensitivity	Specificity	95% CI	P-value
Height (cm)	0.589	74.50 cm	0.203	0.805	0.398	0.532–0.643	0.002
WBC (× 10^9^/L)	0.740	16.10 × 10^9^/L	0.414	0.654	0.760	0.213–0.307	< 0.001
Lymphocyte (× 10^9^/L)	0.641	3.16 × 10^9^/L	0.236	0.571	0.665	0.307–0.411	< 0.001
Monocyte (× 10^9^/L)	0.812	1.32 × 10^9^/L	0.526	0.834	0.692	0.147–0.229	< 0.001
Eosinophil (× 10^9^/L)	0.723	0.14 × 10^9^/L	0.374	0.600	0.774	0.675–0.772	< 0.001
LMR	0.675	3.15	0.276	0.624	0.652	0.624–0.725	< 0.001
AGR	0.735	1.65	0.359	0.712	0.647	0.218–0.312	< 0.001
PA (g/L)	0.730	113.30	0.384	0.810	0.575	0.222–0.318	< 0.001
ALT (U/L)	0.682	22.05	0.329	0.546	0.783	0.631–0.734	< 0.001
GGT (U/L)	0.763	18.20	0.399	0.639	0.760	0.718–0.808	< 0.001
HCT (%)	0.557	36.25%	0.162	0.819	0.343	0.389–0.498	0.043
PLT (× 10^9^/L)	0.590	346.5 × 10^9^/L	0.149	0.505	0.644	0.537–0.644	0.001
GLB (g/L)	0.610	24.55 g/L	0.173	0.548	0.625	0.557–0.663	< 0.001

**Table 4:** The results of multiple logistic regression.

Variables	Coef	S.E	Wald Z	P
intercept	− 1.1157	0.5818	− 1.92	0.0552
Height ≥ 74.50 cm	1.6299	0.4128	3.95	< 0.0001
WBC ≥ 16.10 × 10^9^/L	− 1.3352	0.3595	− 3.71	0.0002
Lymphocyte ≥ 3.16 × 10^9^/L	− 0.6270	0.4104	− 1.53	0.1266
Monocyte ≥ 1.32 × 10^9^/L	− 1.3812	0.3861	− 3.58	0.0003
Eosinophil ≥ 0.14 × 10^9^/L	1.4161	0.3317	4.27	< 0.0001
LMR ≥ 3.15	0.8204	0.3746	2.19	0.0285
AGR ≥ 1.65	− 0.4109	0.3906	− 1.05	0.2928
PA ≥ 113.30 mg/L	− 1.6272	0.3543	− 4.59	< 0.0001
ALT ≥ 22.05 U/L	0.6440	0.3884	1.66	0.0973
GGT ≥ 18.20 IU/L	1.1363	0.3902	2.91	0.0036
HCT ≥ 36.25%	− 0.2676	0.4108	− 0.65	0.5147
PLT ≥ 346.50 × 10^9^/L	1.0132	0.3482	2.91	0.0036
GLB ≥ 24.55 g/L	0.4893	0.4132	1.18	0.2364

### Evaluations of the predictive model

**Figure 4:**
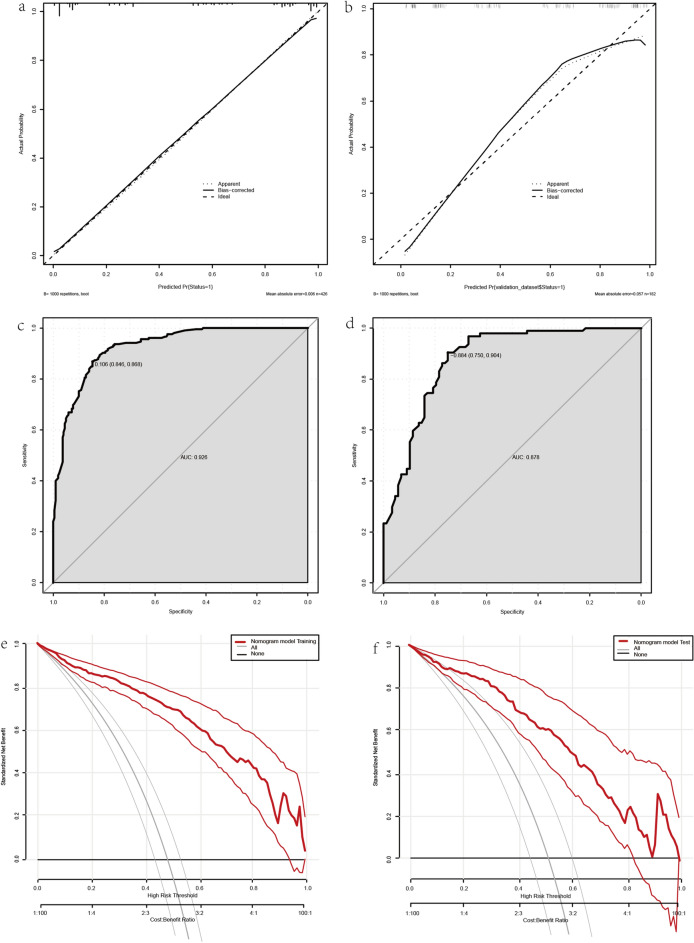
(**a**) Calibration curve of the nomogram in the training set. (**b**) Calibration curve of the nomogram in the validation set. The mean absolute errors of the training and validation set were 0.006 and 0.057. (**c**) Receiver operating characteristic (ROC) curves of the nomogram in the training set. (**d**) ROC curves of the nomogram in the validation set. The sensitivity and specificity of the training set were 0.868 and 0.846. Respectively, the sensitivity and specificity of the validation set were 0.904 and 0.750. (**e**) Decision curve analysis in the training set. (**f**) Decision curve analysis in the validation set.

We evaluated the performance of the nomogram using calibration and discrimination. We internally validated and calibrated the nomogram through 1000 bootstrap analyses. The calibration curve (Fig. 4a,b) results showed no significant deviation from the fit. The predicted results are in accordance with the results showing that the nomogram has a good prediction. The mean absolute errors of the training and validation set were 0.006 and 0.057. The receiver operating characteristic (ROC) (Fig. 4c,d) curve was used to evaluate the diagnostic value of the selected indicators showing good discrimination ability. The area under the ROC curves for the training and validation set was 0.926 and 0.878, respectively. The DCA of the predicted nomogram (Fig. 4e,f) is shown in the figure, which indicates that the model has better clinical application value and decision-making assistance ability.

## Discussion

Currently, clinicians mainly diagnose KD by clinical manifestations and echocardiography, but no specific laboratory method is available for diagnosis. On the one hand, KD patients do not have typical enough clinical features in the early stage, or parents fail to elaborate on typical symptoms with doctors. On the other hand, sepsis and KD patients have similarities in early clinical manifestations and laboratory tests. In addition, incomplete KD and KDSS are challenging to distinguish from sepsis, which adds some difficulties for clinicians in making a precise diagnosis. However, timely diagnosis and treatment of KD can prevent the occurrence of serious sequelae such as CALs. Therefore, we developed a predictive model for diagnosing KD by comparing the laboratory results and epidemiological data of children with KD and sepsis. The model demonstrates good discrimination, predictive power, and clinical utility and can help pediatricians in the early diagnosis of KD by using data from the children's first visit.

In our study, height was the most important predictor. However, there was no statistically significant difference in BMI between KD and sepsis. According to the result, when the child's height is greater than or equal to 74.5 cm, the child is more likely to have KD. Whether this means the age is closer to 1 year old or older, the child is more likely to suffer from KD is worthy of study. Only limited literature is available on KD below the age of 1 year and little information is available from developed countries on this subject^51^. Tseng CF et al. found that 7.5% of children with KD were under one year of age, while Greta Mastrangelo et al. found that 28.3% of children with KD were under one year of age^52,53^. This shows that the prevalence of KD in children under one year of age is lower than in children over one year of age, which in a sense is consistent with our finding that children with a height greater than or equal to 74.5 cm (close to the height of a one-year-old child) are more likely to develop KD.

KD and sepsis patients have leukocytosis in the acute phase, and our study shows that WBC counts are less elevated in KD patients than in sepsis patients and can act as an independent risk. Several studies have reported leukocytes as a biomarker for the diagnosis of KD. In a case–control study by Liu S. et al., WBC counts ≥ 11.12 × 10^9^/L played a predictive role in differentiating KD from non-KD febrile infectious diseases^54^. In addition, in another article by them, WBC < 19.7 × 10^9^/L played an essential role in helping to differentiate KD from sepsis, which is almost consistent with our conclusion that WBC is minor than 16.1 × 10^9^/L in KD patients^50^. In a retrospective study, patients with sepsis had more WBCs (17.94 ± 10.04 × 10^9^/L) than those with viral infection (10.42 ± 4.21 × 10^9^/L) (p < 0.001)^55^. The above may suggest that the immune mechanism of KD is intermediate between sepsis and viral infectious diseases. In addition, Tian Xie et al. reported that patients with abnormally elevated leukocytes were more likely to develop IVIG resistance for both complete and incomplete KD, and patients with CAL had significantly higher WBC than those without CAL^56^.

In a scoring system developed in Taiwan, the percentage of monocytes can be used as an indicator to differentiate KD from febrile children^57^. Vasculitis in KD is characterized by granulomatous inflammation, and monocytes are involved in its formation. Infiltration of monocytes, abnormal activation of macrophages, and production of inflammatory factors and chemokines are involved in forming vascular lesions^58^. Rowley et al. observed that monocytes/macrophages infiltrated arteritis lesions in the autopsy case of KD^59^. Enhanced expression of toll-like receptor (TLR) 2 on monocytes was found in patients with KD and a mouse model of coronary vasculitis^60^. These suggest a pro-inflammatory role for monocytes in KD. Classical monocytes (CM), intermediate monocytes (IM), and non-classical monocytes (NCM) are the three major subpopulations of human monocytes that play pro-inflammatory, antigen expression, and antiviral roles, respectively^61,62^. CM with high SELL expression was significantly elevated in KD patients^63^. In contrast, IM and NCM subpopulations were significantly elevated in sepsis patients^64^. These showed that monocytes might play different roles in the pathogenic mechanisms of KD and sepsis.

Eosinophils are immune cells responsible for allergic reactions and parasite infections^65^. In our study, the eosinophil count was higher in KD than in sepsis and was a significant independent predictor for establishing a diagnostic model. Chih-Min Tsai et al. showed that the percentage of eosinophils (> 1.5%) was the most important independent predictor in a scoring system to differentiate KD from febrile infection, as did Liu Xiaoping Liu et al.^54,57^. In the last decade, eosinophils have been identified as potential sepsis biomarkers. Abidi and Shaaban's Colleagues successively revealed eosinophils’ feasibility and sensitivity in diagnosing sepsis^66,67^. Eosinophil-associated T-helper (Th2) 2 mediators (IL-4, IL-5 and eotaxin) were increased after IVIG treatment, while Th2 is known to play an anti-inflammatory role^68^. In addition, it has been shown that KD patients with an increase in eosinophils have a decreased likelihood of being IVIG resistant and CALs formation^68,69^. These suggest that eosinophils may play an anti-inflammatory role in KD. Patients with KD had higher eosinophil counts before and after IVIG treatment than those with enterovirus^70^. The differences in eosinophil counts between KD and sepsis may indicate that the mechanisms of inflammatory responses in KD and sepsis are different. Also, studies had shown that patients in the KD group had significantly lower peripheral blood eosinophils than the incomplete KD group, which may help diagnose incomplete KD^71^.

Our study found that PA was a predictor compared to ALB. In contrast, the study by Liu et al. collected data only on ALB, one of our innovations^50^. Huang et al. found similarly to us that PA was more valuable than ALB for diagnosing KD, although both ALB and PA levels are reduced in KD patients^72,73^. Relative to ALB, PA has a shorter half-life and is more stable and sensitive than ALB in measuring liver function and malnutrition^74,75^. Research has shown that PA levels in the serum are associated with the prognosis of various diseases^76–78^. Li Zhang et al. found that PA has the following characteristics: (1) A reference value for healthy individuals can be established; (2) It changes significantly in KD patients; (3) IVIG treatment successfully returned to the almost average level^79^. Therefore, it can be used as a marker for the diagnosis and treatment monitoring of KD and the responsiveness to IVIG treatment. Lower AGR and hypoalbuminemia have been identified as independent predictors of CAL^73,80–82^. More specifically, the 22nd Japanese KD Epidemiological Survey revealed that a 1 g/dL reduction in ALB implied a 0.66-fold elevated risk of coronary artery dilation and a 0.34-fold increased risk of the coronary aneurysm^83^. However, these investigators did not collect PA-related information, and perhaps in the future, they may find a more significant role for PA relative to ALB in predicting the occurrence of CALs in patients with KD.

Vasculitis due to KD can involve all medium-sized arteries and viscera, including the liver^84^. Liver pathology in patients with KD can be found with inflammatory cell infiltrates, Kupffer cell augmentation and/or swelling, fatty degeneration and stasis in the sinusoidal and portal vein regions^85,86^. In addition to CALs, hepatic insufficiency is a common manifestation during the acute phase of KD, as evidenced by elevated serum liver enzymes, bilirubin and reduced ALB^87,88^. 90.95% of children with KD presented with at least one liver function indicator abnormality, according to a retrospective study by Goshgar Mammadov and colleagues^80^. Natural killer cells are activated by cytokines, accumulate in inflammatory lesions, and converge on the vascular endothelium and hepatic sinusoids, resulting in hepatocellular injury and endothelial damage. These may be the causes of abnormal liver function in patients with KD^89^. Tremoulet et al. found that 62.7% of KD patients had increased GGT values and 40.3% had ascending ALT values^90^. A predictive model that differentiated KD from febrile illness in Taiwan found that ALT was more specific than aspartate aminotransferase (AST). However, GGT was not included in the study^57^. Nomograms established in the United States and Taiwan show that PLT and ALT are biomarkers for distinguishing KD from febrile disease^57,91^. In our study, according to statistical analysis, GGT was more specific than ALT in diagnosing KD.

An abnormal increase in PLT count is a feature of KD. A retrospective study showed that leukocytes, PLT, CRP, PCT, and other inflammatory mediators were remarkably increased in serum during the acute stage of KD^57^. Activation of PLTs is the first step when blood vessels are damaged, and the endothelium ruptures. In the meantime, PLTs are inflammatory effector cells involved in a range of events from acute inflammation to adaptive immunity^92^. Many receptors on the surface of PLTs frequently interplay with WBCs and endothelial cells. In vitro studies have shown that neutrophils partially depend on PLTs to potentiate fibrin deposits in the blood^93^. These all indicate the correlation between PLTs and vascular inflammation. Unlike Liu's study, our study found that PLTs were an independent predictor of distinguishing KD from sepsis^50^. According to a study in Sichuan, China, other parameters of PLTs, like mean platelet volume and platelet distribution width during fever, can help distinguish KD from other febrile infectious diseases^94^. More studies are needed in the future to verify whether PLT parameters can be helpful in the diagnosis of KD. Furthermore, PLTs and their other parameters may also help diagnose patients with IVIG resistance. In the study of Gang Li et al., thrombocytopenia (< 300 × 10^9^/L) was significantly associated with IVIG resistance in KD patients^95^. Liu et al. suggested that the PLTs reduction in the KD patients with IVIG resistance may be related to the persistent depletion of PLTs due to coronary artery disease^96^. Recently, peripheral biomarkers of immunity/inflammation, neutrophil to lymphocyte count ratio (NLR), and PLR were identified as significant prognostic factors in KD patients with IVIG-resistant^97–101^.

Combined with previous studies on this topic, this article is the first known dynamic nomogram to aid clinicians in differentiating KD from sepsis. Due to using a continuous scale to calculate the probability of a specific outcome, this nomogram has higher accuracy and better identification than other clinical prediction tools or scoring systems. Moreover, this study added the patient's data (height, weight, age, BMI), easy to obtain but easier to ignore in clinical practice. In addition, we added biomarkers such as PA, GGT, LMR and E, which are rarely used to diagnose KD, and appeared to be more specific than ALT and ALB in our study.

However, our study has limitations: (1) Our study is a single-center retrospective article and lacks external validation, so selection bias cannot be ignored; (2) We did not collect data on PCT, IL-6, and erythrocyte sedimentation rate, which increased during the acute episode of KD and sepsis but were not included in the biochemical data. (3) The limitation of lasso regression is that it can drop one reasonably arbitrarily when two independent variables are highly correlated. We will reduce these limitations through further randomized controlled studies and additional external validation.

It is the first time to use a dynamic nomogram to develop a new predictive model that uses height, WBC, monocyte, eosinophil, LMR, PA, GGT, and PLT to help clinicians distinguish KD from sepsis accurately and efficiently.

## Methods

### Study population and design

Medical records of patients admitted to Anhui Provincial Children's Hospital, a 1350-bed tertiary teaching hospital, from January 2020 to May 2021 were retrospectively analyzed. We compared the epidemiological data (including age, gender, height, weight, and BMI) and laboratory characteristics of KD patients with those of sepsis. The inclusion criteria for our study were: (1) patients diagnosed with KD, including classic KD, incomplete KD, and KDSS; (2) patients diagnosed with sepsis; (3) patients were younger than 10 years old; The exclusion criteria for our study were: (1) patients received any IVIG or steroid therapy in the month prior to the laboratory test; (2) patients with autoimmune disease; (3) patients with congenital cardiovascular disease; (4) patients with blood disease; (5) patients diagnosed with septic shock; (6) patients with incomplete clinical data. Although the study was conducted during the Covid-19 pandemic, no patients were diagnosed with Multi-System Inflammatory Syndrome in Children (MIS-C) in our hospital. Therefore, our study did not include MIS-C patients.

### Data collection

We collected data on 38 variables from epidemiological data, routine blood data, and biochemical test data. Epidemiological data include age, sex, height, weight, and BMI. Routine blood data includes WBC, neutrophil, lymphocyte, monocyte, eosinophil, RBC, hemoglobin (HB), HCT, mean vascular volume (MCV), mean corpuscular hemoglobin concentration (MCHC), RDW, PLT, NLR, LMR, and PLR were also calculated from routine blood data. Biochemical tests data includes total bilirubin (TBIL), direct bilirubin (DBIL), indirect bilirubin (IBIL), TP, ALB, globulin (GLB), AGR, PA, ALT, AST, alkaline phosphatase (ALP), GGT, lactate dehydrogenase (LDH), BUN, Na, Ca, iron (Fe), and CRP. All data were collected at the first visit before IVIG administration in patients with KD and before antibiotic treatment in patients with sepsis.

### Definitions of KD and sepsis

The diagnosis of KD was made according to the 2017 American Heart Association (AHA) criteria^12^. The diagnosis of classic KD is based on a fever ≥ 5 days and fulfilling at least 4 of the 5 main clinical features. The five clinical features include: (1) Erythema and cracking of the lips, strawberry tongue, and/or erythema of the oral and pharyngeal mucosa; (2) Bilateral bulbar conjunctival congestion without exudate; (3) Rash: maculopapular, diffuse erythrodermic, or erythema multiform; (4) Erythema and edema of the hands and feet in the acute phase and/or periungual desquamation in the subacute phase; (5) Enlarged cervical lymph nodes (≥ 1.5 cm in diameter), usually unilateral. Incomplete KD is diagnosed by a fever of more than 5 days with 2 or 3 consistent major clinical features and ≥ 3 additional laboratory findings or positive echocardiogram. Additional laboratory findings include anemia for age, PLT ≥ 450,000 after day 7 of fever, ALB ≥ 3.0 g/dL, increased ALT, WBC ≥ 15,000/mm^3^, WBC/HPF ≥ 10 on urinalysis. Sepsis was defined in accordance with the 2016 Surviving Sepsis Campaign Guidelines^102^. Sepsis is diagnosed by signs and symptoms of inflammation and infection with hyperthermia or hypothermia (rectal temperature of 38.5 or 35 °C), tachycardia (which may not be present in hypothermic patients), and signs of altered function in at least one of the following organs: altered mental status, hypoxemia, increased serum lactate levels, or bounding pulses.

### Statistical analyses

All continuous variables were not normally distributed after the normality test. We used the Wilcoxon rank sum test to analyze the quantitative variables. The Chi-square test and Fisher's exact test were applied to analyze the categorical variables. We randomly sampled the entire sample thousand times to build a prediction model that can help differentiate the KD from sepsis. We randomly selected 70% of the patients for the training set. Then the other 30% of the patient data was used for the testing set. Secondly, the significant variables are the intersection of LASSO and SVM. The ROC converted the selected continuous variables to the categorical variables when the AUC value was maximum. Thirdly, we built a prediction nomogram using a multiple logistic regression model to show each predictor's odds ratios and β factors. Data analysis was achieved by R software, version 4.1.2. P-values < 0.05 were considered statistically significant. Finally, we use the shiny platform to build a dynamic nomogram.

### Guidelines and regulations statements

All methods were carried out in accordance with relevant guidelines and regulations.

### Ethics statements

This study was conducted in accordance with the Declaration of Helsinki. This study involving human participants were reviewed and approved by The Medical Research Ethics Committee of Anhui Provincial Children’s Hospital (No.EYLL-2022-028) (in the supplementary materials S1).

### Patient consent

This retrospective study was approved by the Medical Research Ethics Committee of Anhui Provincial Children’s Hospital (No. EYLL-2022-028). The informed consent from a parent and/or legal guardian was waived, which is approved by the Medical Research Ethics Committee of Anhui Provincial Children’s Hospital.

## Supplementary Information

Supplementary Table 1.

## Data Availability

The datasets used and/or analyzed during the current study are available from the corresponding author upon reasonable request.

## Abbreviations

KD: Kawasaki disease
CAL: Coronary artery abnormalities
IVIG: Intravenous immunoglobulin,
WBC: White blood cell
CRP: C-reactive protein,
PCT: Procalcitonin
AHA: American Heart Association,
KDSS: Kawasaki disease shock syndrome
WBC: White blood cell count
NLR: Neutrophil to lymphocyte count ratio
PLR: Platelet to lymphocyte count ratio
LMR: Lymphocyte to monocyte count ratio
RBC: Red blood cell count
HB: Hemoglobin
HCT: Hematocrit
MCV: Mean corpuscular volume
MCHC: Mean corpuscular hemoglobin concentration
RDW: Red blood cell distribution width
PLT: Platelet count
TBIL: Total bilirubin
DBIL: Direct bilirubin
IBIL: Indirect bilirubin
TP: Total protein
ALB: Albumin
GLB: Globulin
AGR: Albumin/globulin
PA: Prealbumin
ALT: Alanine aminotransferase
AST: Aspartate aminotransferase
ALP: Alkaline phosphatase
GGT: γ-Glutamyltransferase
LDH: Lactate dehydrogenase
BUN: Blood urea Nitrogen
Na: Sodium
Ca: Calcium
Fe: Iron
LASSO: Least absolute shrinkage and selection operator
SVM: Support vector machine
ROC: Receiver operating characteristic,
AUC: Area under the curve
DCA: Decision curve analysis
TLR: Toll-like receptor
CM: Classical monocytes,
IM: Intermediate monocytes
NCM: Non-classical monocytes,
Th2: T-helper 2
TTR: Transthyretin
