# Supplementary material for: A machine learning model for distinguishing Kawasaki disease from sepsis
Source: Sci Rep. 2023 Aug 2;13:12553. doi: 10.1038/s41598-023-39745-8 (PMC10397201; doi:10.1038/s41598-023-39745-8)
Supplement: Supplementary file 1 — Supplementary Table 1. [file 41598_2023_39745_MOESM1_ESM.docx]

**Table S1. The Baseline Characteristics between the KD and Sepsis Groups**

| **Variables** | **sepsis** | **KD** | **P** |
| --- | --- | --- | --- |
| **Age (months)** | 25.95 ± 25.01 | 28.37 ± 21.69 | 0.001 |
| **Height(cm)** | 84.88 ± 21.09 | 88.5 ± 16.56 | <0.001 |
| **Weight(kg)** | 12.95 ± 7.14 | 13.43 ± 4.79 | <0.001 |
| **BMI (kg/m^2^)** | 17.29 ± 3.19 | 16.96 ± 2.47 | 0.462 |
| **WBC(×10^9^/L)** | 21.5 ± 8.83 | 15.25 ± 5.46 | <0.001 |
| **N(×10^9^/L)** | 14.41 ± 7.42 | 10.46 ± 4.89 | <0.001 |
| **L(×10^9^/L)** | 5.09 ± 4.77 | 3.57 ± 2.11 | <0.001 |
| **M(×10^9^/L)** | 2.3 ± 7.77 | 0.92 ± 0.49 | <0.001 |
| **E(×10^9^/L)** | 0.11 ± 0.2 | 0.31 ± 0.42 | <0.001 |
| **NLR** | 4.49 ± 4.74 | 4.22 ± 3.55 | 0.437 |
| **PLR** | 90.7 ± 70.66 | 133.66 ± 84.74 | <0.001 |
| **LMR** | 3.3 ± 3.15 | 4.59 ± 3.04 | <0.001 |
| **RBC (×10^12^/L)** | 4.24 ± 0.51 | 4.09 ± 0.41 | <0.001 |
| **HB (g/L)** | 110.69 ± 14.84 | 108.26 ± 10.83 | 0.004 |
| **HCT (%)** | 34.51 ± 3.96 | 33.34 ± 3.32 | <0.001 |
| **MCV (fL)** | 80.79 ± 6.6 | 81 ± 5.21 | 0.850 |
| **MCHC(g/L)** | 321.67 ± 15.04 | 325.07 ± 15.08 | 0.020 |
| **RDW (%)** | 11.15 ± 2.68 | 11.9 ± 2.78 | 0.002 |
| **PLT (×10^9^/L)** | 321.27 ± 121.47 | 367.01 ± 139.1 | <0.001 |
| **TBIL (umol/L)** | 8.24 ± 5.42 | 10.49 ± 11.88 | 0.282 |
| **DBIL (umol/L)** | 3.43 ± 2.42 | 5.82 ± 9.71 | 0.097 |
| **IBIL (umol/L)** | 4.81 ± 3.35 | 4.67 ± 2.92 | 0.860 |
| **TP (g/L)** | 63.75 ± 7.25 | 62.16 ± 6.62 | <0.001 |
| **ALB (g/L)** | 40.62 ± 4.89 | 36.84 ± 4.26 | <0.001 |
| **GLB (g/L)** | 23.45 ± 4.68 | 25.32 ± 5.35 | <0.001 |
| **AGR** | 1.87 ± 1.34 | 1.51 ± 0.31 | <0.001 |
| **PA (g/L)** | 122.34 ± 45.81 | 89.39 ± 30.35 | <0.001 |
| **ALT (U/L)** | 28.86 ± 81.56 | 72.22 ± 120.42 | <0.001 |
| **AST (U/L)** | 42.27 ± 123.55 | 62.68 ± 137.65 | 0.676 |
| **ALP (U/L)** | 146.93 ± 46.62 | 148.19 ± 72.68 | 0.088 |
| **GGT (U/L)** | 21.01 ± 36.39 | 68.23 ± 89.53 | <0.001 |
| **LDH (U/L)** | 316.05 ± 268.43 | 284.12 ± 98.72 | 0.040 |
| **BUN (mmol/L)** | 4.07 ± 3.63 | 4.36 ± 16.08 | <0.001 |
| **Na (mmol/L)** | 136.59 ± 3.26 | 135.75 ± 2.77 | <0.001 |
| **Ca (mmol/L)** | 2.77 ± 5.78 | 2.34 ± 0.15 | <0.001 |
| **Fe (mmol/L)** | 3.13 ± 2.3 | 3.17 ± 2.3 | 0.041 |
| **CRP (mmol/L)** | 85.44 ± 57.59 | 80.56 ± 56.46 | 0.136 |
| **Gender** |  |  | 0.123 |
| **Female** | 123 (39.8%) | 100 (33.4%) |  |
| **Male** | 186 (60.2%) | 199 (66.6%) |  |
